# Psychosocial interventions for autistic children and adolescents delivered by non-specialists in low- and middle-income countries: a scoping review

**DOI:** 10.3389/fpsyg.2023.1181976

**Published:** 2023-08-07

**Authors:** Megan Cherewick, Christina Daniel, Catherine Canavan Shrestha, Priscilla Giri, Choden Dukpa, Christina M. Cruz, Roshan P. Rai, Michael Matergia

**Affiliations:** ^1^Department of Community & Behavioral Health, Colorado School of Public Health, University of Colorado Anschutz Medical Campus, Aurora, CO, United States; ^2^Department of Biostatistics & Informatics, Colorado School of Public Health, Aurora, CO, United States; ^3^Darjeeling Ladenla Road Prerna, Darjeeling, West Bengal, India; ^4^Department of Psychiatry, School Psychology Program, University of North Carolina at Chapel Hill School of Medicine, Chapel Hill, NC, United States; ^5^Broadleaf Health & Education Alliance, Stroudsburg, PA, United States; ^6^Center for Global Health, Colorado School of Public Health, Aurora, CO, United States

**Keywords:** autism spectrum disorder, child, adolescent, psychosocial, low- and lower-middle-income countries, non-specialist, mental health

## Abstract

**Background:**

Most autistic individuals reside in low- and middle-income countries (LMIC) and have limited access to medical providers and specialists. Support for delivery of psychosocial interventions by non-specialists is growing to address this mental health care gap. This scoping review involved a systematic analysis of studies of non-specialist delivered psychosocial interventions for children and adolescents diagnosed with autism and living in low- and middle-income countries.

**Methods:**

The primary objective of this review was to identify psychosocial interventions for autistic children and adolescents in LMIC delivered by non-specialists (parent, teacher, peer, community, multi-level) and to summarize resulting effects on targeted outcomes. The search strategy was completed in four databases with predetermined inclusion and exclusion criteria. The systematic search generated 3,601 articles. A total of 18 studies met inclusion/exclusion criteria. Data extraction was completed, and results summarized by; (1) participant sample; (2) intervention procedures; (3) implementation by non-specialists; (4) effect on evaluated outcomes; and (5) assessment of risk of bias. Studies examined a range of child and adolescent outcomes including assessment of communication skills, social skills, motor skills, functional and adaptive behaviors, emotional regulation, attention and engagement, sensory challenges, depression, anxiety, and quality of life. Several studies also evaluated intervention effects on family relationships, parent/caregiver stress and parent/caregiver mental health.

**Results:**

Collectively, the 18 studies included a total of 952 ASC participants ranging in age from 2 to 16 years. Of the included studies, 8 studies were parent/caregiver-mediated, 1 study was peer-mediated, 2 studies were teacher-mediated, and 7 studies included multi-level non-specialist mediated components. Effects on evaluated outcomes are reported.

**Conclusion:**

Non-specialist delivered interventions for autistic children and adolescents are effective for an array of outcomes and are particularly well suited for low- and middle-income countries. Implications for future research are discussed.

## 1. Introduction

Autism Spectrum Disorder (ASD) refers to a group of neurodevelopmental disorders characterized by differences in communication, socialization and repetitive or restricted patterns of behaviors, interests, or activities (American Psychiatric Association, 2013). These characteristics present differently in each autistic child and include a range of strengths and challenges. Classification of autism as a disorder in the DSM-5 nomenclature has received increasing attention. The term disorder motivates alignment with deficit focused frameworks and subsequent treatment approaches. More recently, autistic individuals and advocates have supported redefinition of Autism Spectrum Disorder to Autism Spectrum Condition (ASC) to highlight heterogeneity in presentation that includes both challenges and strengths associated with autism. In this article, we will use the term “autism” or ASC to refer to autistic children and adolescents.

Autism Spectrum Condition has a global prevalence rate estimated to be between 0.7 and 1.5%, making ASC one of the most common developmental disabilities (Baird et al., 2006; Lord and Spence, 2006; Fombonne, 2009; Lyall et al., 2017). Comorbidity of ASC with other mental health challenges is common, including anxiety, depression, externalizing behaviors, attention deficit hyperactivity disorder, feeding disorders, sleep disorders and sensory processing disorder which (Kogan et al., 2008; LoVullo and Matson, 2009; Reaven, 2009; Hodgetts et al., 2015; Vohra et al., 2017). A common challenge are sensory processing differences, estimated to be present in 80% of children with ASC (Ben-Sasson et al., 2009). Another study found 50% of parents described their autistic child as having more than four comorbid problems (Mannion et al., 2013).

Caregivers of autistic children often experience financial impacts related to caring for their child and may be unable to earn a livelihood due to the responsibilities of caring for their child without adequate support (Rogge and Janssen, 2019). While it is difficult to compare costs associated with ASC globally due to wide variation in cost categories by country, the total costs of care are profound regardless of context. Costs include caregiver productivity loss, medical care, special education, specialized therapies, and accommodation in residential facilities (Amendah et al., 2011; Deanna and Dana, 2011; Buescher et al., 2014). In recent reviews it was estimated that overall lifetime costs for autistic individuals are estimated to be $2.4 million – 3.2 million in US dollars with costs of services accounting for 79% of the total cost burden (Ganz, 2007; Järbrink, 2007; Buescher et al., 2014; Rogge and Janssen, 2019).

In the past few decades, new approaches to supporting autistic children – predominantly psychosocial interventions, have been developed. Psychosocial interventions are interpersonal or informational activities, techniques, or strategies that aim to improve the health, functioning and wellbeing of children by targeting biological, behavioral, cognitive, emotional, social, or environmental factors that affect autism outcomes (Committee on Developing Evidence-Based Standards for Psychosocial Interventions for Mental Disorders et al., 2015). While traditional approaches to “treating” autism have focused on deficits and inabilities, a paradigm shift toward neurodiversity frameworks shifts attention toward recognition of differences in abilities *and* strengths. Common strengths of autistic children include excellent memory skills, attention to detail, motivation to recognize patterns, visual learning, analytical proficiency, and creative thinking (Craig and Baron-Cohen, 1999; Baron-Cohen, 2006; Chamak et al., 2008; Dawson and Mottron, 2009; Mottron et al., 2013, 2014; Russell et al., 2019). A neurodiversity framework combines recognition of differences in functional and behavioral presentation, and strengths, to center intervention focus on inclusion, needed accommodations and support tailored to each autistic child. Current research has reached consensus that autistic children require *specialized* interventions *tailored* to support needs to address challenges in communication (Smith et al., 2004), social interaction (Mcconnell, 2002), sensory regulation (Dawson and Watling, 2000; Baranek, 2002), and behaviors (Matson and Rivet, 2008).

Of the estimated 52 million individuals with ASC in the world, most autism research has been completed in high-income countries (HIC) even though most autistic children live in low- and middle-income countries (LMIC) (Rahman et al., 2016; Pervin et al., 2022). While there is growing evidence for the positive impact of psychosocial interventions, the vast majority have been developed and tested in HIC in the global north although 95% of autistic individuals live in LMIC without access to diagnostic, treatment or support services (Pervin et al., 2022). For example, South Asia is a region with the largest number of children in the world with recent epidemiological estimates from India indicating approximately two million families had a child with ASC between the ages of 2–9 (Deshmukh et al., 2013). A recent meta-review analyzing systematic reviews on the effectiveness of interventions in autistic children and adolescents compared intervention approaches reported from HIC and LMIC (Pervin et al., 2022). Results from the meta-review included 35 systematic reviews; 6 included comprehensive treatment programs addressing multiple developmental domains (e.g., communication skills, social skills, daily living skills, and sensory regulation), two of which were from LMIC; 14 included focused interventions targeting a specific behavioral or developmental problem such as joint attention, five of which were from LMIC; and 11 reviews included complementary and alternative medicine interventions (e.g., acupuncture, massage, herbal medicine), seven of which were from LMIC (Handleman and Harris, 2001; Vismara and Rogers, 2008; Pervin et al., 2022). Results also reported that 15 reviews included delivery by non-specialists with 5 from LMIC; 4 reviews included medical interventions with two from LMIC; and 15 reviews examined technology-assisted interventions, two from LMIC (Vismara and Rogers, 2008).

To address the care gap for autistic children in LMIC, the global community has begun to consider different implementation strategies to deliver psychosocial support that is feasible, acceptable, and sustainable in resource-poor settings. The body of research produced in high-income countries on autism is an important contribution to the evidence in design of effective autism interventions, however, these findings need to be translated appropriately with communities to be effective in contexts where resources and cultural belief systems vary dramatically. An opportunity for innovation in LMIC to develop, test, and refine new intervention approaches exists that can advance progress toward supporting development of autistic children and adolescents that is effective, efficient, scalable, and sustainable in LMIC.

Seizing this opportunity will require addressing cultural knowledge, beliefs, and behaviors specific to a particular setting, as well as more universal challenges such as access to formal diagnostic evaluation, time, and costs associated with programs for autistic individuals. A crucial difference between HIC and LMIC contexts is the availability of specialist providers. Interventions in HICs most often include parent coaching and one-on-one therapy with speech language pathologists, occupational therapists, physical therapists, developmental interventionists, and developmental and psychiatric physicians (Rojas-Torres et al., 2020; Gibson et al., 2021; Kumar et al., 2022). In low-resource contexts, lack of access to mental health specialists is limited and specialists are typically concentrated in urban areas and these specialists are more likely to expect higher salaries provided by private sectors which can further exacerbate the gap between poorer and more affluent communities to afford and access needed services.

An additional barrier faced in LMIC is the limited availability of specialists that can provide a clinical diagnosis of ASC. Many of the “gold standard” diagnostic tools are difficult to access by researchers in LMIC and/or may not be available in local languages, culturally adapted or validated in LMIC. The costs associated with using these tools are estimated to exceed *per capita* annual healthcare expenditures for most of the global ASC population by more than four-fold (Durkin et al., 2015). Heterogeneity in the presentation of autism poses challenges to developing tools with the sensitivity and specificity to capture the full range of presentation of autism symptoms across the spectrum (Durkin et al., 2015). In addition, cultural differences in perceptions of typical or atypical behavior are interwoven with culturally defined norms and standards (Mandell and Novak, 2005; Norbury and Sparks, 2013; Donohue et al., 2019; de Leeuw et al., 2020). For example, measures evaluating eye contact and imaginary play are commonly used to screen and diagnosis autism in the global north, but in other contexts, these measures may not be well-aligned to cultural value systems (Zhang et al., 2006; Bernier et al., 2010; Bornstein, 2013; Smith et al., 2017). While there has a been a proliferation in screening and diagnostic tools, comparatively less research studies have been published testing ASC interventions in LMIC (Rojas-Torres et al., 2020).

Within the field of global mental health, task-shifting approaches have been utilized to address human resource challenges associated with the limited availability of specialists (Divan et al., 2015; Matergia et al., 2019). In task shifting, professionals train and coach non-accredited human resources (such as lay counselors and community health workers) to deliver care. Examples of this approach for child and adolescent mental health may be particularly instructive given the co-morbidity of autism and other mental health challenges. In LMIC settings where psychologists and therapists are in short supply, there is emerging evidence for the delivery of mental health care by lay counselors such as teachers. For autistic children and adolescents, non-specialist mediators of psychosocial interventions have included parents, teachers, peers and/or community members.

### 1.1. Objectives

A review of psychosocial interventions for autistic children in LMIC, delivered by non-specialists is important to build a nuanced evidence-base that examines which outcomes were attained by non-specialists. The primary objective of this review was to evaluate current evidence on use of non-specialist delivered interventions for autistic children and adolescents in low- and middle-income countries. This review sought to describe the characteristics of studies, type of non-specialists delivering interventions; effect on evaluated targeted outcomes, and to appraise the certainty of the evidence by completing a risk of bias (RoB) quality assessment. A secondary objective was to identify potential synergies for impact by evaluating effects of multi-level implementation designs to identify gaps in the existing research and to inform future research efforts. This paper adds to the literature in providing a comprehensive overview of psychosocial interventions and the effect on outcomes delivered by non-specialists in LMIC.

## 2. Methods

### 2.1. Systematic search procedures

The initial search strategy was designed with assistance from a health sciences librarian at CU Anschutz Strauss Medical Libraries to search the following: (1) Medical Subject Headings (MeSH) terms for autism spectrum disorder, (2) MeSH terms for child OR adolescent, (3) key words for psychosocial intervention studies, and (4) LMIC status determined by World Bank-defined low-and middle-income countries. The search was completed on December 5th, 2022. The following data bases were queried: MEDLINE ALL (1946 to date before search date); Embase (^1^1974 to search date); Web of Science, American Psychological Association PsychInfo (1806 to present), and Global Index Medicus (World Health Organization). We identified additional sources through bibliography scans of included references. Search terms were first developed for Ovid Medline and were subsequently translated for each database (Table 1).

**TABLE 1:** Systematic search strategy terms.

Category	Search Terms	Search Fields
Condition	Autis*, Aspberger*, neurodisabilit*	Title or abstract
Age	Adolescen*, teen*, youth*, young people, young adult, early adulthood, schoolchild, child*, kid*, juvenile*, toddler*, minor*, pediatric*, boy*, girl*	Title or abstract
LMIC	LMIC*, limited resource setting*, resource poor setting*, low resource setting*, developing countr*, developing nation*, less developed, underdeveloped, low income, middle income, global south, country name^+^	
Mental health and psychosocial intervention	Mental health services, psychotherapy, community mental health services, psychological techniques, social work, social support, self-help, primary prevention, secondary prevention, tertiary prevention, mental hygiene services, psychosocial support system, psych*, psychosocial, counseling, intervention, therapy, psychoeducation, structured activities	Anywhere in article

^+^All World Bank defined low- and middle-income country names were included in the search strategy.

### 2.2. Inclusion and exclusion criteria

All raters screened title and abstracts and applied the inclusion/exclusion criteria displayed in Table 2. Inclusion criteria included: (1) target population was children or adolescents (defined as ages 0–19), (2) target population was children/adolescents who had received an autism diagnosis, (3) included original data resulting from a psychosocial intervention that was delivered by non-specialists, (4) conducted in an LMIC, and (5) were published in English up until December 5th, 2022, (6) study sample > 10. Psychosocial interventions were defined as “interpersonal or informational activities, techniques, or strategies that target biological, behavioral, cognitive, emotional, interpersonal, social, or environmental factors with the aim of improving health functioning and well-being” (Committee on Developing Evidence-Based Standards for Psychosocial Interventions for Mental Disorders et al., 2015). Non-specialists were defined to include community health workers, parents/caregivers, teachers, community leaders, and peers. Studies were excluded if they did not meet the inclusion criteria. Three authors examined articles for eligibility (CD, CCS, and MC).

**TABLE 2:** Inclusion and exclusion criteria.

Domain	Inclusion Criteria	Exclusion Criteria
Language	English language	Not in English language
Publication type	Published and peer-reviewed articles	Chapters from books Study protocols Systematic Reviews Scoping Reviews Guidelines and clinical protocols Advocacy documents Unpublished dissertations Conference abstracts/presentations
Publication date	1806–2022	N/A
Research setting	Low- or middle-income country	High income country
Sample	Sample includes only or mainly children and/or adolescents under the age of 19 who had received a diagnosis of autism spectrum disorder (ASC) Sample size>10	Sample contains only adults Sample size <10
Study Design	Feasibility or pilot randomized controlled trials; randomized controlled trials	Epidemiological studies Cross-sectional studies Case studies
Intervention evaluated	Psychosocial intervention targeting ASC children or adolescents Intervention delivered by non-specialists	Intervention delivered by specialist professionals Pharmacological treatment Biomedical treatments Robot or artificial intelligence treatment

### 2.3. Inter-rater agreement

Title and abstract screening agreement using the inclusion and exclusion criteria was obtained in 95.12% of studies. During full-text review, agreement between reviewers was obtained in 74.19% of studies reviewed. Discrepancies were resolved through discussions among CC, CCS and MC.

### 2.4. Quality assessment procedures

Quality assessment was completed using the Revised Cochrane risk-of-bias tool for randomized trails (RoB 2) and were completed independently by two review authors (SG and CD) with discrepancies resolved by MC (Sterne et al., 2019). Table 3 reports the risk of bias domains; (1) Sequence generation; (2) Allocation concealment; (3) Blinding of participants, personnel and outcome assessors; (4) Incomplete outcome data; (5) selective outcome reporting; and (6) Other sources of bias (Sterne et al., 2019). Judgements were rated as ‘high’, ‘moderate’ and ‘low’ risk of bias.

**TABLE 3:** Revised Cochrane risk of bias tool (Sterne et al., 2019).

Domain	Description
Sequence generation: Was the allocation sequence adequately generated?	Describe the method used to generate the allocation sequence in sufficient detail to allow an assessment of whether it should produce comparable groups.
Allocation concealment: Was allocation adequately concealed?	Describe the method used to conceal the allocation sequence in sufficient detail to determine whether intervention allocations could have been foreseen in advance of or during enrollment.
Blinding of participants, personnel, and outcome assessors: Was knowledge of the allocated intervention adequately prevented during the study?	Describe all measures used, if any, to blind study participants and personnel from knowledge of which intervention a participant received. Provide any information relating to whether the intended blinding was effective.
Incomplete outcome data: Were incomplete outcome data adequately addressed?	Describe the completeness of outcome data for each main outcome, including attrition and exclusions from the analysis. State whether attrition and exclusions were reported, the numbers in each intervention group (compared with total randomized participants), reasons for attrition/exclusions where reported, and any re-inclusions in analyses performed by the review authors.
Selective outcome reporting: Are reports of the study free of suggestion of selective outcome reporting?	State how the possibility of selective outcome reporting was examined by the review authors, and what was found.
Other sources of bias: Was the study apparently free of other problems that could put it at a high risk of bias?	State any important concerns about bias not addressed in the other domains in the tool. If questions/entries were pre-specified in the review’s protocol, responses should be provided for each question/entry.

## 3. Results

### 3.1. Sources of evidence

**Figure 1:**
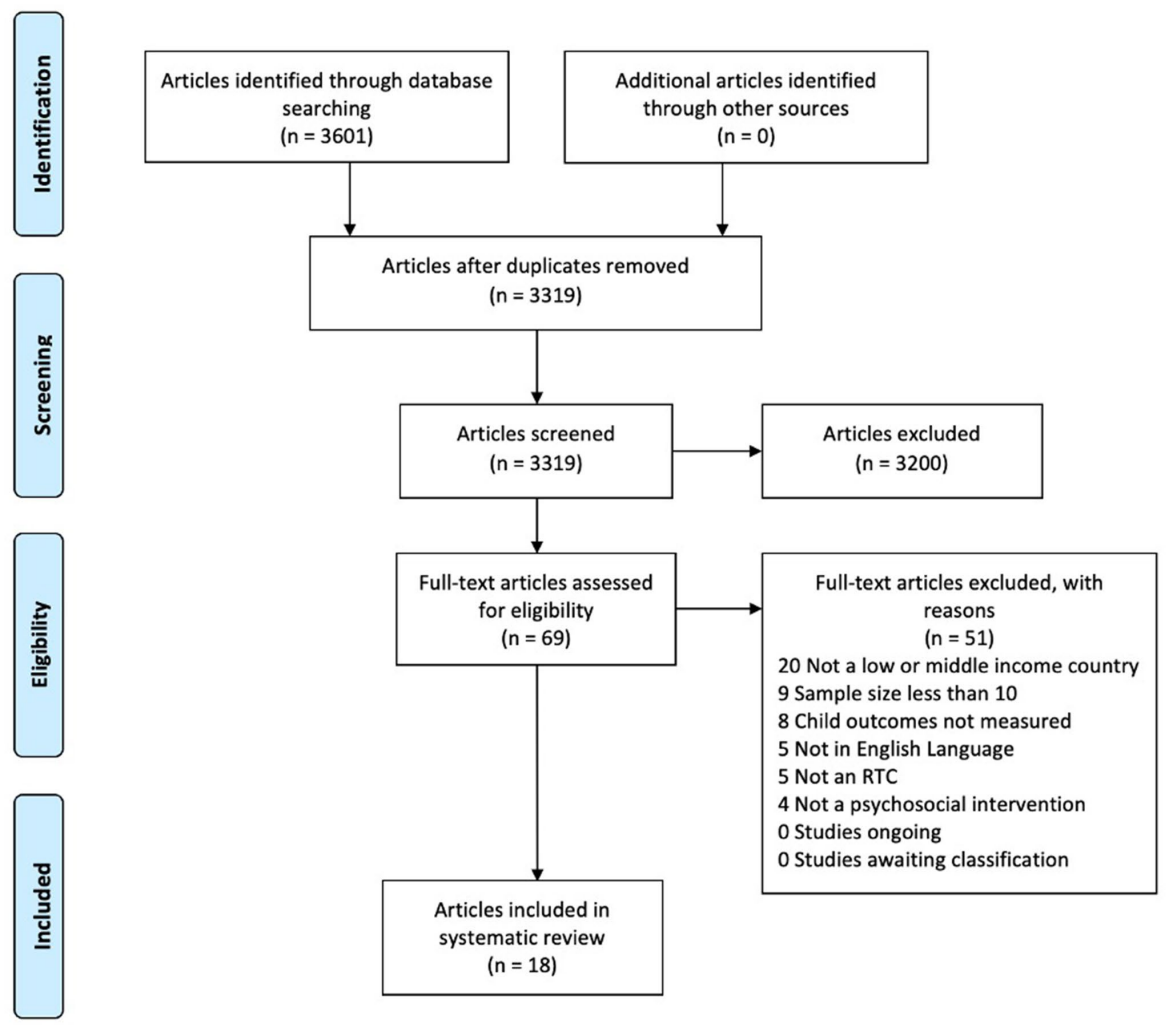
Preferred reporting items for systematic reviews and meta-analyses (PRISMA) flow diagram (Moher et al., 2009).

After completing the search, 3,877 total citations were retrieved. Duplicates were removed and organized using the citation management software Endnote version 20. After duplicates were removed 3,601 total unique citations were uploaded to Covidence, a systematic review citation and screening software. Covidence detected 282 additional duplicates leaving 3,319 citations for initial title/abstract screening. After screening titles/abstracts, 3,200 articles were excluded for not meeting the inclusion/exclusion criteria. Full-text review was completed on the remaining articles (*N* = 69) and studies were excluded if they were found not to meet the inclusion criteria. A total of 18 articles were included in the final review and reasons for excluded studies (*N* = 51) are presented in the PRISMA systematic review flow diagram is presented in Figure 1.

### 3.2. Quality assessment procedures

Quality assessment was completed using the Revised Cochrane Risk-of-Bias tool for randomized trails (RoB 2) and were completed independently by two review authors (SG and CD) (Sterne et al., 2019). Final judgements of risk of bias were then reached by consensus (SG, CD and MC). In total, 8 studies were assessed to have low risk of bias; 7 were judged to have ‘moderate’ risk of bias; and 3 were assessed as “high” risk of bias.

### 3.3. Data synthesis

A scoping review database was created in Covidence to allow for extraction of data from each of 18 empirical research articles. All studies included participants who had received an autism diagnosis. The following data was collected from each study: (1) bibliographic information including author, year, country, study design; (2) sample size and description; (3) intervention descriptions and outcomes targeted; (4) implementation methods, challenges, and successes; and (5) main findings on effect on targeted outcomes. Detailed results of included studies, effects on measured outcomes, and risk of bias judgements are presented in Table 4 and organized by type of non-specialist delivered components. In total, 952 autistic children and adolescents were included in the 18 studies. Eight included studies were parent/caregiver mediated approaches that were sometimes supplemented by coaching of parents by therapists. In some studies, the intervention was designed to integrate educational and developmental techniques such as those from Developmental, Individual-Difference, Relationship-Based DIR techniques (Pajareya and Nopmaneejumruslers, 2011; Casenhiser et al., 2015), Early Start Denver Model (Dawson et al., 2010), Early intensive behavioral intervention (Smith, 2010), parent coaching (Ingersoll and Dvortcsak, 2019), Naturalistic developmental behavioral interventions (Schreibman et al., 2015), and Home-based sensory intervention (Chara and Chara, 2004; Pfeiffer et al., 2011).

**Table 4:** Data extraction results of included studies (*N* = 18).

Citation	Country	Participant Age	RCT Design	Intervention Description	Implementation Notes	Outcome Measures	Effect on Outcomes	Risk of Bias
	SES	Sample Size	Non-Specialist			Assessor		
Akhani et al. (2021)	Iran	3–5 years old	Control group received medical, occupational and speech therapy	Frequency: weekly Intensity: Six 2-h group sessions; Six 1-h individual Duration: 12-weeks Adapted from Ingersoll and Dvortcsak (2019) parent training protocol; floor time approach to social engagement and imitation, child-led strategies, prompting and rewarding behaviors, and special interests	-COVID-19 quarantine disrupted therapeutic services -increase in economic hardship, social isolation	Gilliam Autism Ratings Scale (GARS); Functional-Emotional Assessment Questionnaire; Function Assessment Scale; Parenting Stress Index-Short Form; WHO Quality of Life-Brief (WHOQL)	+Global autism symptoms +Functional emotions +Quality of life –Parenting stress	Low
	GDP: 4388 POV: 1.0	*N* = 40	Parent/Caregiver			Evaluation specialists		
Bordini et al. (2020)	Brazil	3–6 years old	Single-blinded; Control received standard community treatment	Frequency: weekly Intensity: 90-min sessions Duration: 22-weeks Parent training and video modeling based on ABA discrete trial teaching, errorless learning and prompting of hierarchy of approach (most intrusive help to independence)	Limited to families with DVD player Parents not blinded to treatment assignment	Vineland Adaptive Behavior Scale (VABS); Snijders-Oomen Nonverbal Intelligence Test (SON); Autism Behavior Checklist (ABC); Hamilton Depression Rating Scale (HDRS)	+Communication +Non-verbal IQ n.s. global autism symptoms	Low
	GDP: 8917 POV: 5.8	*N* = 67	Parent/Caregiver			Psychologists and Psychiatrists		
Ho and Lin (2020)	Taiwan	3–5 years old	Control group received routine treatment as usual	Frequency: weekly Intensity: 2-h weekly sessions for 2 weeks; 15-h per week thereafter Duration: 14-weeks Developmental individual-difference relationship-based model (DIR); encouraged child-initiated activities. Week 1: parent completes course to learn basics; Week 2: Parents learn specific techniques; Week 3: Parents practice techniques Weeks 4–14: Parents implemented program with monthly support	Neither parent groups achieved the suggested intensity of 15 h per week	Childhood Autism Rating Scale (CARS2-ST); Functional Emotional Assessment Scale (FEAS); Chinese version of psychoeducational profile – third edition (CPEP-3); Vineland Adaptive Behaviors Scale (VABS)	+Self-regulation +Social engagement +Communication +Motor adaptive skills +Parent–child interactions n.s. adaptive behaviors	Low
	GDP: 12720 POV: 0.1	*N* = 24	Parent/Caregiver			Occupational therapist (CARS2-ST); Parents/caregivers (FEAS; VABS)		
Leung et al. (2016)	China	2–5 years old	Parallel RCT	Frequency: weekly Intensity: 2-h sessions Duration: 8-weeks Parental coaching program in communication, social emotional skills, and behavioral management	Only the intervention group assessed for maintenance effects	Eyberg Child Behavior Inventory (ECBI); Parenting Stress Index/Short Form (PSI/SF); Parenting Scale (PS)	-Behavioral problems -Parenting stress -Dysfunctional discipline strategies	Low
	GDP: 12720 POV: 0.1	N = 115	Parent/Caregiver			Parents/Caregivers		
Liang et al. (2022)	China	Less than 4 years	Control group waitlisted	Frequency: weekly Intensity: Eight 2–3-h group training sessions; 2 individual trainings for 0.5 h every 2 weeks Duration: 12-weeks Parent coaching on parent–child relationships; behavioral strategies, sensory stimuli, body and object use, speech/language, relational skills, social and self-help skills	Focus on cognitive abilities and behavioral problems	Autism Behavior Checklist (ABC); Childhood Autism Rating Scale (CARS); Gesell Developmental Schedule (GDS)	+Adaptive behavior, +language +Social behavior -behavioral problems n.s. fine/gross motor	Low
	GDP: 12720 POV: 0.1	*N* = 70	Parent/Caregiver			Parents/Caregivers		
Malucelli et al. (2021)	Brazil	2–4 years old	Control group received treatment as usual	Frequency: weekly Intensity: 2 h weekly Duration: 12-weeks Parental Coaching based on Early Start Denver Model (ESDM); identified goals and strategic plans to address goals; focus on parent/child interaction; videos taken by parents for analysis	–Mall sample not representative of diverse population	Early Start Denver Model Curriculum Checklist; Denver Model commitment coding sheet	+ Receptive/expressive communication +Social capacity +fine/gross motor skills, + independence +Parent/child interaction quality	Med
	GDP: 8917 POV: 5.8	*N* = 18	Parent/Caregiver			Early Start Denver Model-certified professionals		
Manohar et al. (2019)	India	2–6 years old	Parallel RCT; Control group received treatment as usual	Frequency: weekly Intensity: n/a Duration: 12 weeks Developmental Behavioral Interventions (NDBI) based parent coaching on joint attention skills, verbal and motor imitation, social engagement, and adaptive skills	High parent fidelity High acceptability of intervention.	Parent-Rated 10-Item Likert Scale (PRILS-10); Children’s Global Assessment Scale (CGAS); Pediatric Quality of Life Inventory 4.0 (PedsQL); Short Sensory Profile-2	+Social skills +Non-verbal communication –Parental stress +Parental coping n.s. verbal communication n.s. object engagement	Med
	GDP: 2388 POV: 10.0	*N* = 50	Parent/Caregiver			Parents/Caregivers (PRILS-10) and Short Sensory Profile-2 and PedsQL; Research investigators: (CGAS)		
Padmanabha et al. (2019)	India	3–12 years old	Parallel group pilot RCT Control group received speech therapy and ABA	Frequency: 5 days per week Intensity: 45–60 min sessions Duration: 12-weeks Pre-designed structured Home-Based Sensory Intervention (HBSI) including tactile, proprioceptive, vestibular, visual, and auditory stimulation; Parents given training, a manual, and training videos.	Not double blind	Children’s Global Assessment Scale (CGAS); Pediatric Quality of Life Inventory (PedsQL); Short Sensory Profile-2; Childhood Autism Rating Scale (CARS); Vineland Social Maturity Scale (VSMS); Parent-Rated 10-Item Likert Scale (PRILS-10)	– Sensory challenges – Hyperactivity – Stereotypic behavior + Overall wellbeing + Cognition	Med
	GDP: 2388 POV: 10.0	*N* = 40	Parent/Caregiver			Parents/Caregivers (PRILS-10) and Short Sensory Profile-2 and PedsQL; Researcher investigators (CGAS and VSMS)		
Zhang et al. (2022)	China	4–12 years old	Single-blind parallel RCT; Control group received ABA therapy	Frequency: 3 times per week Intensity: 40-min sessions Duration: 2-months Neurotypical peers facilitate active participation in play and social interactions and provide positive feedback	Peers did not work well with ‘severely autistic’ children	Social Responsiveness Scale (SRS); Childhood Autistic Rating Scale (CARS)	+Social skills –Global autism symptoms	Low
	GDP: 12720 POV: 0.1	*N* = 55	Peer			Therapists completed assessments		
Fazlioglu and Baran (2008)	Turkey	7–11 years old	Control group attended regularly scheduled special education classes	Frequency: 2 times per week Intensity: 45-min sessions Duration: 12-weeks Program targets 13 behaviors based on “The Sensory Diet,” A schedule of frequent and systematically applied somatosensory stimulation.	Not reported	Sensory Evaluation Form for Children with Autism	– sensory challenges +Auditory responsiveness – stereotypic behavior +Social communication	High
	GDP: 10616 POV: 0.4	*N* = 30	Teacher			Physiotherapist		
Ji and Yang (2021)	China	12–13 years old	Control group received psychological counseling	Frequency: 3 times per week Intensity: 1-h sessions Duration: 6-weeks Virtual environment (VT) training; Physical exercise (PE) through football training	Not reported	Multiple Object Tracking (MOT) for assessing visual attention	+Visual attention	High
	GDP: 12720 POV: 0.1	*N* = 100	Teacher			Researchers		
Nnamani et al. (2019)	Nigeria	15–16 years old	Waitlist control group	Frequency: 2 times per week Intensity: 2-h sessions Duration: 12-weeks Rational Emotive Behavior Therapy: family members serve as adolescent’s communication and social partners	Inclusion criteria limited to English speaking families with 2 participating neurotypical family	Autism communication and social skills scale for adolescents (ACSSSA);	+Communication skills +Social skills	Med
	GDP: 2184 POV: 30.9	*N* = 68	Parent and Sibling			Participants		
Ireri et al. (2019)	Kenya	5–12 years old	Block randomized RCT; Control group did not receive treatment	Frequency: weekly Intensity: Thirteen 1-h individual sessions; 7 group sessions Duration: 6-months Multimodal Anxiety and Social Skills Intervention grounded in CBT delivered across three modalities: individual, group and family/school	Did not account for potential confounders such as use of medication, social contact, or adult attention	Social Responsiveness Scale (SRS-2); Child and Adolescent Symptom Invesntory-4 ASC Anxiety Scale (CASI-Anx)	+Social skills – Anxiety	High
	GDP: 2099 POV: 29.4	*N* = 40	Parent and Teacher			Parents/Caregivers		
Kanagaraj et al. (2022)	India	4–10 years old	Parallel-group RCT; Control group received Cognitive Behavioral Therapy	Frequency: weekly Intensity: 1-h sessions Duration: 6-months Emotional enhancement intervention (EEI) and Cognitive Behavioral Therapy; picture exchange communication, video modeling of primary emotions and expressive storytelling, emotional modeling	Extended duration and period of follow up	Gilliam Autism Rating Scale (GARS-3); Indian Scale for Assessment of Autism (ISAA)	+Adaptive behavior +Social skills +Emotional reciprocity	Med
	GDP: 2388 POV: 10.0	N = 34	Parent & Teacher			Research investigators supported by 3 clinical psychologists		
Piravej et al. (2009)	Thailand	3–10 years old	Block randomized RCT; Control group received sensory-integration therapy only	Frequency: 2 times per week Intensity: Two 1-h sessions per week Duration: 8-weeks Sensory-integration therapy and Thai traditional massage	No assessment of child–parent relationships	Conners’ Teacher Questionnaire; Conners’ Parent Questionnaire; Scale; Sleep Diary (SD)	+Sleep behaviors +Conduct problems – Anxiety	Med
	GDP: 6908 POV: 0.0	*N* = 60	Parent and Teacher			Parents/Caregivers; Teachers		
Xu et al. (2018)	China	2–5 years old	Control group received speech therapy and occupational therapy, sensory integration, music/dance, and play therapy	Frequency: 5 days per week Intensity: 1-h sessions Duration: 8-weeks Early Start Denver Model (ESDM); teacher led training of parents to support parent–child interactions	No assessment of fidelity	Childhood Autism Rating Scale (CARS); Autism Behavior Checklist (ABC); Chinese version of Autism Treatment Evaluation Checklist (ATEC)	– Global autism symptoms n.s. global autism symptoms	Med
	GDP: 12720 POV: 0.1	*N* = 36	Parent and Teacher			Clinicians		
Divan et al. (2019)	India	5 years old	Control groups received allopathic or homeopathic care	Frequency: biweekly Intensity: 1-h sessions Duration: 6-months Social communication intervention based off PASS; Facilitated Play based therapy with video feedback; inclusion of child’s special interests in directing play	Sample size limits power to evaluate effect	Brief Observation of Social Communication Change (BOSCC); Dyadic Communication Measure for Autism (DCMA); Vineland Adaptive Behavior Scale (VABS); Parental Mental Wellbeing (PHQ-9); Research on Autism and Families in India (RAFIN)	+Communication +Parent mental health n.s. global autism symptoms	Low
	GDP: 2388 POV: 10.0	*N* = 40	Parent and Community			Research Investigators; Parents/Caregiver (PHQ-9 and RAFIN)		
Rahman et al. (2016)	India	2–9 years old	Single-blind RCT; Control group received treatment as usual	Frequency: biweekly Intensity: 1-h session Duration: 6-months PASS communication intervention: one on one home sessions to scaffold communication skills; delivered by community health workers and parents	Did not evaluate comorbidities, sleep, or behavioral problems	Dyadic Communication Measure for Autism (DCMA); Vineland Adaptive Behaviors Scale (VABS); MacArthur Communicative Development Inventory; Communication and Symbolic Behavior Scales Development Profile	+Communication –Joint attention n.s. language acquisition n.s. adaptive behaviors	Low
	GDP: 2388 POV: 10.0	*N* = 65	Parent and Community			Research Investigators (DCMA); Parents/Caregivers		

SES, socioeconomic status as reported by the World Bank (2022) including Gross Domestic Product (GDP) per capita in US dollars and Poverty headcount ratio (POV) reported as the percentage of the population earning less than $2.14. +, positive significant effect; –, negative significant effect; n.s., non-significant.

One study was a peer-mediated intervention to improve social interaction. Two studies were teacher-mediated with one using virtual reality training and physical education and the other focused on sensory regulation based on “the Sensory Diet” (Lease et al., 2016). A total of seven included studies included a parent component and a second level; one study was a parent and sibling mediated intervention, four were parent and teacher mediated interventions, and two were parent and community mediated interventions. The parent-sibling-mediated intervention adapted Rational emotive behavior therapy (Ellis and Dryden, 2007) Parent-teacher-mediated interventions including adaptations of Multimodal anxiety and social skill intervention (White et al., 2013), emotional enhancement intervention (Solomon et al., 2004), massage therapy, and the Early Start Denver Model (Dawson et al., 2010). The parent-community-mediated interventions were both modeled after parent-mediated interventions in India (Rahman et al., 2016). The duration of intervention in included studies ranged from 3 weeks to 6 months with considerable variation in the intensity of sessions (daily to weekly). The control conditions reported ranged from no treatment, or ‘treatment as usual’ to alternative treatments.

## 4. Discussion

### 4.1. Justification for the need to leverage non-specialists in LMIC

Investment in capacity building around training professionals to meet the needs of children with autism may not be the most efficient or effective approach in LMIC. First, the costs associated with training professionals can be resource intensive and time-consuming. Lessons learned from LMIC indicate that it can be difficult to incentivize trained professionals to serve populations in remote, rural, locations (Strasser, 2003; Pruitt and Epping-Jordan, 2005, Anyangwe and Mtonga, 2007). Second, trained professionals are also more likely to expect higher salaries provided by private sectors which can further exacerbate the gap between poorer and more affluent communities to afford and access needed services (Kolehmainen-Aitken, 2004; Hagopian et al., 2009). Third, the types of supportive therapy that may be effective in ASC children and adolescents is widely variable and comparative studies between specialists and non-specialists delivering interventions have not been completed. Autism interventions must be flexibly adapted to the unique sets of needs and strengths of each autistic child, and non-specialists close to autistic individuals may be best positioned to notice these needs and strengths (Fleming et al., 2015). Non-specialists such as parents, teachers, peers, siblings and community members can identify opportunities to scaffold learning approaches that reinforce or inclemently challenge a child appropriately (Burrell and Borrego, 2012; Guralnick, 2019). Fourth, a focus on delivery of therapeutic interventions by specialists can dissuade education systems from embracing inclusive learning environments that have been shown to be effective in supporting learning of a broad array of neurodiverse students (Mariga and McConkey, 2014). Fifth, leveraging naturalistic, real-life settings is important for supporting the development of ASC children by providing opportunities to practice and master skills in different settings. This may be particularly true for ASC where the disabilities experienced by differences in social emotional and communication areas are experienced in relationship with the context in which the individual lives (e.g., neighborhoods, schools, work environments) (Ebbels et al., 2019).

### 4.2. Parent/caregiver mediated interventions

Most autism intervention approaches in low- and middle-income countries are parent/caregiver mediated interventions (*N* = 8; 44%). In total, Parent/Caregiver intervention approaches included children ages 2–12, with all but one study targeting children ages 2–6. The reason for targeting parents/caregivers during this age is based on evidence suggesting positive effects of early intervention (ages 1–7) on developmental trajectories (Anderson et al., 1987; Corsello, 2005; Itzchak and Zachor, 2011), however the quality of the evidence base is low with few successful replication studies (McConachie and Diggle, 2007). Results from meta-analyses of parent-mediated RCTs have found small but significant effects for parent–child interaction only, with insignificant effects for communication, language, adaptive behavior and adaptive behavior (Oono et al., 2013; Nevill et al., 2018). While reviews have reported small positive effects for children, there is evidence of greater improvements for parents, including reduced stress and increased sense of competence (Wyatt Kaminski et al., 2008; Beaudoin et al., 2014). Reduction in parenting stress is an important outcome because parents and caregivers of autistic children experience higher rates of mental health disorders than parents of neurotypical children (Bitsika and Sharpley, 2004; Davis and Carter, 2008; Estes et al., 2009; Phetrasuwan and Shandor Miles, 2009).

A challenge to meta-analysis of parent-mediated interventions is that programs vary dramatically in theoretical background supporting the approaches tested. More recently, critiques of early intervention have emerged that argue that early intervention approaches have often set goals that are not well matched to common autistic learning trajectories and/or target goals that unnecessary for healthy development such as reduction in repetitive behaviors and special interests (Mottron, 2017). While many autism interventions have focused on supporting parents/caregivers of children with ASC to reduce the mental health burden of parents/caregivers who experience stress, financial difficulties, and stigma, more recent research has aimed to capture positive parenting experiences of children with developmental disabilities (Hastings and Taunt, 2002).

### 4.3. Teacher mediated interventions

Two studies reviewed were teacher mediated interventions delivered in school classrooms. One study completed in Turkey included autistic children 7–11 years old who received 12-weeks of the intervention that included somatosensory stimulation based on the “The Sensory Diet” (Fazlioglu and Baran, 2008). This study found significant improvements in auditory responsiveness, and social communication and a significant reduction in repetitive behavior and sensory problems. The second teacher-mediated intervention included autistic children ages 12–13 living in China and included six weeks of virtual environment training and physical exercise with a significant effect reported on visual attention outcomes (Ji and Yang, 2021). Both studies were assessed to have high risk of bias.

Teacher-mediated interventions for autistic children and adolescents are a promising new approach to addressing ASC support services, especially in LMIC settings where special education services are limited, and most school-attending autistic children are in general education classrooms. Many teachers have received training in child development and are skilled in individualizing instruction based on developmental level. By building off this experience, teachers may be trained in the skills needed to support autistic children and adolescents with different need. Furthermore, teachers have daily consistent access to autistic children and can observe changes in behavior, social, emotional, and cognitive patterns. Prior research has indicated that teachers trained in integrating mental health care for indicated cases in delivery of curriculum can benefit children with mental health challenges and simultaneously support the mental wellbeing of classmates (Cruz et al., 2021). While this intervention was not designed for autistic children, a motivation for teachers delivering care in an inclusive classroom environment to empower teachers with the skills needed to address a range of mental health symptoms. Training teachers to support autistic children could similarly help to address a broad range of needs and strengths of autistic children and could potentially help to reduce stigma associated with autism. Enhancing the capacity of teachers to help support learning in an inclusive environment can reduce costs associated with private schools and/or special needs schools and provides an additional benefit of allowing autistic children to learn through peer-modeling of neurotypical kids. Additionally, a recent review on economic costs related to ASC found that education related costs were a major cost component for parents and children (Rogge and Janssen, 2019). Enhancing the capacity of teachers to help support learning in an inclusive environment can reduce costs associated with private schools and/or special needs schools and provides an additional benefit of allowing autistic children to learn through peer-modeling of neurotypical kids (Anaby et al., 2019).

### 4.4. Peer mediated interventions

Peer mediated interventions include approaches where peers are trained to deliver or facilitate programs (Harrell et al., 1997; Laushey and Heflin, 2000). Only one study included in this review was peer mediated. This study was completed in China with autistic children ages 4–12, matched to neurotypical peers trained to support social skill development over 2 months (Zhang et al., 2022). Importantly, while results indicated a significant improvement in social skills, the authors noted that peer interventionists did not work well with ‘severely autistic children’(Zhang et al., 2022). In high-income countries, peer-mediated intervention approaches for ASC have shown similar positive effect on acquisition of social skills (DiSalvo and Oswald, 2002; Bass and Mulick, 2007). Including peers in school-based intervention approaches may be particularly beneficial in combination with teacher-mediated interventions because peers can provide reinforcing opportunities for social emotional learning and to practice social emotional skills in the classroom, during breaks, during mealtimes, during recess, and in before and after school programs (Chan et al., 2009).

Findings from developmental science on neurotypical children suggest that key transitional periods or inflection points during adolescence can improve the efficacy of interventions through precision in timing, sequencing, and design (Crone and Fuligni, 2020). The rapid changes in neural development and hormones that occur during puberty heighten the affective salience of experiences with peers, family, school, and the community. As adolescents develop, they are increasingly sensitive to social status and admiration or social rejection and loneliness (Steinberg, 2008; Blakemore and Mills, 2014; Lam et al., 2014; Shulman et al., 2016; Orben et al., 2020). As adolescents learn to navigate an increasingly complex social world, they actively shape their own identity, sense of purpose, and set goals for the future (Crone and Dahl, 2012; Pfeifer and Berkman, 2018). Integrating peer-mediated components during adolescence, particularly early adolescence (ages 10–14) should be explored for autistic children and including peers in intervention approaches can also reinforce inclusivity in learning environments and could potentially reduce stigma associated with autism.

### 4.5. Multi-level interventions

Seven studies were categorized as ‘multi-level’ for including both a parent-mediated component with a sibling component (*N* = 1), teacher component (*N* = 3), or community member component (*N* = 3). In Nigeria, a parent and sibling intervention included autistic adolescents ages 15–16 that completed a 120 week Rational Emotive Behavior Therapy intervention that reported positive effects on communication skills and social skills (Nnamani et al., 2019). Four studies included parent and teacher-mediated components in Kenya, India, Thailand, and China. Age ranges of included participants ranged from 2 to 12 years of age and duration of the intervention varied from 8-weeks to 6-months. Positive significant effects were found for communication and social skills, adaptive behavior, and improved sleep; significant reduction in global autism symptoms, anxiety and repetitive behaviors were reported. Two included studies involved parent and community member components, both completed in India over 6-months. The community members involved in the interventions were community members who received training. These two studies reported positive effects on communication skills and parental mental health, but non-significant findings were reported for global autism symptoms, verbal language, and adaptive behaviors. For many mental health disorders, psychosocial interventions that engage multiple levels within an individual’s social ecology have proven to be successful (Kohrt et al., 2018).

### 4.6. Implications for future research

A comprehensive review of interventions for autistic children in LMIC is useful because certain types of needs and strengths of autistic children may be most effectively addressed if developmentally timed, sequenced and matched to the support system most effective in affecting change in particular areas of difference (Green, 2019). Leveraging naturalistic, real-life settings is important for supporting the development of autistic children. Scaffolding appropriate opportunities for mastery and challenge in different contexts and with different influential people within the social-ecology of autistic children should be explored (Carey et al., 2019). In all contexts, but particularly in LMIC comprehensive approaches addressing multiple developmental domains are most promising because they can better address the diverse needs and strengths of autistic individuals in comparison to focused treatment approaches targeting a specific need.

In addition to matching non-specialist mediated interventions to developmental periods, future research should consider the strength and quality of existing evidence and the perspectives of autistic adults on which outcomes should be targeted, when and by whom. Findings from developmental neuroscience are useful to consider which outcomes to target during a developmental period. For example, experience-expectant learning theories explain that neurological development influences whether or not the brain can process and learn from environmental stimuli (Greenough et al., 2002). Further, research with autistic adults has underscored the situational importance of whether particular autistic traits are advantageous or disadvantageous in different contexts because particular traits may be more or less favorably perceived dependent on cultural and situational factors (Mandell and Novak, 2005; Norbury and Sparks, 2013; Donohue et al., 2019; de Leeuw et al., 2020). For example, eye contact may be valued in some cultural contexts but may be unexpected or even perceived as disrespectful in others (Zhang et al., 2006; Bernier et al., 2010; Bornstein, 2013; Smith et al., 2017).

Meta-analyses show that specialist interventions have been completed mostly in HIC to address behavioral problems, however, it is plausible that comprehensive treatment approaches beginning during early childhood and continuing through early adulthood delivered by non-specialists may prevent the need for clinical intervention. Comparative evaluation studies examining differences in implementation strategies and effectiveness are needed to identify mechanistic pathways through which interventions achieve effects. Implementation science is urgently needed to identify mechanisms of change that result in positive effects. Mechanisms of change include affective, behavioral, cognitive, and physiological mediating variables (Cherewick and Matergia, 2023). Identification of implementation barriers and facilitators would provide evidence needed to understand the implementation factors required to achieve effects in diverse contexts. Implementation science can also support identification of essential elements to achieve effects, thereby eliminating redundant or ineffective elements and reshaping intervention design strategies to focus on amplifying the most potent elements of interventions.

**Figure 2:**
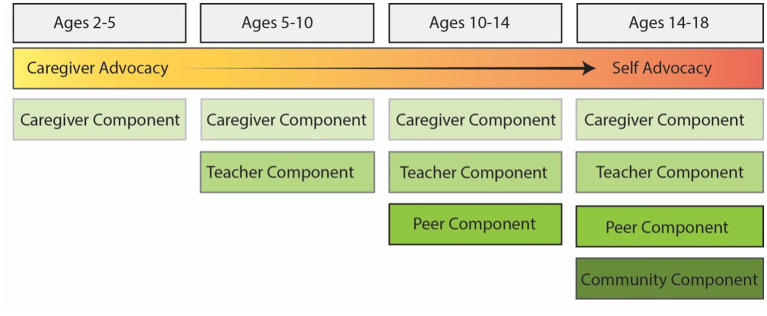
Developmentally sequenced social ecological intervention model for autistic children and adolescents.

Future research should consider testing interventions during developmental periods where non-specialist interventionists are likely to be most influential in supporting needs and leveraging strengths of autistic children. A general schema for sequential and multi-level intervention components is provided in Figure 2. Caregiver mediated interventions may be best matched to support communication and regulation skills during early development. Teacher mediated interventions may be best matched to support executive function, and adaptive behavior. Peer mediated interventions may be best matched to supporting social emotional learning. Community mediated interventions may be best matched to for mastery learning in specific vocational skills. Across development, integrating positive, neurodiversity affirming advocacy messages may be especially beneficial for autistic children and adolescents. Designing effective advocacy focused components that can be integrated into interventions should consider participatory methods and co-design with autistic adults.

The addition of an overarching advocacy component across childhood and adolescence in Figure 2 is included to represent the reinforcing potential of advocacy efforts that begin with caregivers and transition to self-advocacy as autistic children develop. Autism advocacy can reduce stigma, and allows autistic children and their families to articulate needed accommodations and strengths (Mitter et al., 2019). Previous research has explored participatory methods and co-design of autism interventions with autistic adults (Chamak et al., 2008; Davidson, 2010; Kuper et al., 2021). Autistic adults can bring lived experience to improve all aspects of the research process including design of studies, intervention, implementation, and evaluation methods. Future research should consider inclusion of autistic adolescents in design of intervention programming. Inclusion of autistic individuals in design of interventions may also help support flexibility in implementation by identifying how interventions can be best tailored to different developmental levels, needs, and strengths.

### 4.7. Limitations

Due to time and resource restraints, this study was limited to randomized controlled trial designs. We recognize that other study designs are important to designing effective interventions. We excluded studies that used artificial intelligence and/or robot implementation methods because these methods did not fit our pre-determined definition of psychosocial interventions. However, technology-mediated interventions and those that use artificial intelligence, while nascent, may hold promise in the future.

## 5. Conclusion

Non-specialist mediated interventions for autistic children and adolescents are well suited for resource poor-environments. Studies included in this review demonstrated non-specialist delivered interventions in LMIC had positive effects in communication/language, social skills, motor skills, adaptive behaviors, and improved mental wellbeing. Moreover, synergies between non-specialist mediated intervention approaches across development should be matched and sequenced to developmental periods. An approach that engages multiple non-specialists in a child’s social ecological environment can be particularly beneficial to allow autistic children and adolescents to practice and master learning in different settings. Multi-level intervention approaches can also effect change in the individuals’ delivering interventions, leading to enhanced autism advocacy efforts to reduce stigma and celebrate the unique strengths of autistic individuals.

## Data availability statement

The original contributions presented in the study are included in the article/supplementary material, further inquiries can be directed to the corresponding author.

## Author contributions

MC, MM, and CC conceived of the objective of the systematic review. MC developed the search strategy and completed searches in databases. CDa and CS completed title and abstract screening, and full text-reviews. CDa and MC completed quality assessments of included articles. MC resolved any conflicts. MC, MM, RR, CDa, PG, CC, CDu, and CS analyzed and synthesized the results. All authors contributed substantially to writing the manuscript, read and approved of the manuscript prior to submission.

## Conflict of interest

The authors declare that the research was conducted in the absence of any commercial or financial relationships that could be construed as a potential conflict of interest.

## Publisher’s note

All claims expressed in this article are solely those of the authors and do not necessarily represent those of their affiliated organizations, or those of the publisher, the editors and the reviewers. Any product that may be evaluated in this article, or claim that may be made by its manufacturer, is not guaranteed or endorsed by the publisher.

## Footnotes

^1^ Embase.com
